# A Philosophical and Clinical Treatise on Ophthalmology, Based on the Principles of the Dynamic Therapeutics

**Published:** 1846-01

**Authors:** 


### ART. XI.

*Traité philosophique et clinique d'Ophthalmologie, basé sur les principes de la Thérapeutique Dynamique.* Par M. F. ROGNETTA, Docteur en Médecine et en Chirurgie, &c. &c.

*A Philosophical and Clinical Treatise on Ophthalmology, based on the principles of the Dynamic Therapeutics.* By F. ROGNETTA, M.D. *Paris,* 1844. 8vo, pp. 724.

THIS is a second and enlarged edition of Dr. Rognetta's 'Cours d'Ophthalmologie,' published in 1839, and forms a complete systematic treatise on the disorders of the eye and its appendages, in which the author does not give merely the results of his own experience, but analyses and criticises the writings of his predecessors in the same field. Without losing sight of the grand object of such a work, the rendering it as practically useful as possible, Dr. Rognetta has by no means evaded the discussion of disputed points in pathology and practice. On the contrary, he presents us with a lively and interesting account of the doctrines of all the celebrated ophthalmologists of the present age; and when he states his own opinion, assigns his reason for their adoption with a clearness and frankness which are peculiarly pleasing. We have no hesitation in recommending the work, as being not only one of the most minute, but also one of the most readable books on eye-diseases which we have met with, containing a vast amount of information, collected with great pains, put together in an honest, manly manner, and written in a clear and agreeable style.

As for the claim which Dr. Rognetta sets up for his book, as being

"philosophical and clinical," we cannot say so much. The school of Rasori, to which the Doctor belongs, seem one and all to fancy themselves philosophers. We might let this pass; but to assume the title "philosophical" as peculiarly appropriate to their system, and as distinguishing it from all other medical systems, like the assumption of "physiological" by the school of Broussais, is more apt to make them appear ridiculous than to excite respect. Neither can we admit Dr. Rognetta's work as a clinical one, any more than other ophthalmological treatises in which numerous cases are detailed, but these chiefly borrowed from the writings of other authors, few of them personally and publicly treated by him, who, collecting them from various sources, illustrates by their aid the different topics which comes before him.

The chief peculiarity by which the work of Dr. Rognetta is distinguished is, that the author is a follower of the school of Rasori and Giacomini, from the pathological and therapeutical principles of which he professes never to swerve.

Our readers are sufficiently aware, that the doctrine of Rasori differs from the Brownonian chiefly in this, that while Brown considered every influence and substance in nature, capable of acting on the living frame, as a stimulant, Rasori supposes a numerous class of substances to act as direct sedatives, and they serve to depress the morbid excitement which may happen to exist in the organs of the body. Remedies, then, of this class Rasori calls *contro-stimulants.* Contrary to Brown, he regarded all diseases as sthenic; but from this doctrine Giacomini has departed, admitting diseases of debility, and in this he is followed by Rognetta, who refers (p.8,) to the ophthalmoid as examples of the sthenic class of diseases, in which "the morbid state of the living tissues is such that their vital force, their sensibility, their dynamism, in a word, is exalted," while as examples of the asthenic class he refers to certain of the amauroses, met with in those exposed to certain poisonous influences, such as the vapours of lead, mercury, &c., or debilitated by chronic hemorrhagies, the abuse of certain remedies, and the like,—causes which act upon the whole economy, and produce a morbid condition, which, like the former, is entirely functional or vital, and consists in a deficiency of stimulus.

By way of introduction, Dr. Rognetta gives a sketch of what may be termed *ophthalmic therapeutics.* As this is the most original part of his work, and contains numerous statements well deserving of attention, it will be chiefly to it that our remarks shall be directed.

He begins by observing, that since the time of Broussais, attention has been much concentrated on the study of the character of diseases. Hence the art of diagnosis has made immense progress, while the science which treats of those phenomena which follow the administration of medicines, and precede the restoration of health, has been almost completely neglected. A belief seemed to have gained ground, (but the remark must be understood as applying chiefly to France,) that the whole science of medicine consisted in pathology, and that therapeutics should follow pathological knowledge as an immediate corollary, which it would be sufficient to announce purely in a general way. To understand thoroughly the action of medicines was regarded as superfluous, and, with the exception of bleeding, abstinence, some few emollients, and certain substances regarded as possessing a calming property, medical applications were abjured as irritants and incentives. To judge of the action of medicines fell, as an accessory province, into the hands of mere naturalists and chemists. The former Dr. Rognetta accuses of minding only the local effects of medicines, and excluding from consideration the influence which they exercise after their passage into the circulation; the latter, of neglecting the dynamic action of medicines, their action, that is to say, on the vital powers, and of too often supposing the living body to resemble the retorts and other apparatus of a chemical laboratory.

"To judge," exclaims he, "of the action of medicines, we must study their dynamic effects, according to the changes which they produce in the functions, and not according to their local effects, which can conduct only to false conclusions—a study new to France, imported from Italy by M. Mojon and myself, in our translation of the important work of M. Giacomini.* In a dynamic point of view, the knowledge of the therapeutical action of substances is altogther independent of chemistry, and belongs to the domain of physiological observation and of clinical experience. Chemistry has to do merely with the preparation of medicines, not with their action." (p. 14.)

Dr. Rognetta proceeds, in chapters fifth and sixth, to a review of the general and local medications used in the cure of the diseases of the eye. The former, he says, have been classed chiefly under three heads, viz. tonics, antiphlogistics, and revulsives; while others have been considered as specifics. This last class, as well as the three former, he merges into one or other of the two divisions under which he comprehends all ophthalmic medications, viz. hypersthenisants and hyposthenisants, in other words, stimulants and counter-stimulants, or excitants and sedatives.

1. Under the head of *tonics* or *excitants*, Dr. Rognetta throws out a number of objections to the vulgar use of these terms, and to their indiscriminate application not merely to wine, brandy, and the like, but also to the acids, bark, nux vomica, gentian, iron, and zinc, substances which have few common properties to bring them under one class.

"As the strength of the body depends on the integrity of the functions, whatever remedy re-establishes these in the healthy condition may be called a tonic. Bleeding will be a tonic, if it removes a pneumonia or an ophthalmia, and thus restores the lungs or the eye to its state of natural strength; and the same with tartar emetic, and a crowd of other antiphlogistic remedies. The patient is weak, it is remarked of one affected with scrofulous corneitis or chlorotic amaurosis; he requires tonics. He is weak, but why is he weak? Undoubtedly from the same cause that he is ill. Now, it is not by administering stimulants to him that his strength will be restored, but by re-establishing his organic functions; and if iron, or iodine, or bark attain this object, surely it is not by exciting the system.

"There are very few substances really capable of raising the rhythm of the functions, or rather the organic force, above the normal type, or above the level at which it may be at the time. They are alcoholics, opium, cinnamon, and the others. These are true excitants, but not tonics.

"The term *tonic* ought to be abandoned, or employed only in a wide sense, to signify a means capable of re-establishing the functions. Such a means is not always an excitant, nor are excitants always tonics. Thus, if alcohol, wine, or cinnamon be given to an ophthalmic patient, weak from scrofula, chlorosis, or intermittent fever, he will be rendered still weaker; the vital force, no doubt, or the organic sensibility will be raised above its former level, but, at the same time,

* Traité Philosophique et Expérimental de Matière Médicale et de Thérapeutique.

the normality of the functions, upon which strength depends, will not be restored." (pp. 17-19.)

Bitter substances are accounted tonics, as bark, coffee, hops, chicory, burdock, chamomille, bitter almonds, orange peel, angelica, &c., but the assertion, according to Dr. Rognetta, is altogether arbitrary and false. Far from being excitants, these substances are hyposthenisants, not because they are bitter, but because experience has shown this to be their dynamic action. Strange to tell, the only bitter substances the action of which is really excitant, such as opium, are not placed under this category. The reader must not, then, be surprised, when he finds the bitters prescribed by our author with a different intention from that which is usually assigned to them, or finds condemned those combinations of substances of opposite actions, the mixtures of which must become either totally inert, or of very equivocal efficacy; such as that of calomel with opium, opium with tartar emetic, bark infused in wine, camphor dissolved in alcohol, and the like.

Dr. Rognetta observes that it has been thought that the nature of the dynamic action of a medicine might be changed, by administering it in a larger or smaller dose; that calomel and the acids, for example, might be antiphlogistics in a small dose, and excitants in a large one, and *vice versa* for other medicines. This he regards as an error. So long as the composition of the medicine is not changed, its action cannot be fundamentally different—merely the degree of its energy will vary with the dose. The primitive intrinsic action is invariable also, whatever be the nature of the disease for which the drug is prescribed.

We think our readers will agree with us, that in his remarks on tonics, of which the above extracts and abstract afford a fair sample, there is more truth than novelty, and more puerility than truth. At the same time, the obvious conclusion to be drawn, we think, from the facts stated under this head by Dr. Rognetta, is in favour of more numerous and more minute subdivisions of the materia medica than those which he admits.

2. There are a few affections of the eye, observes Dr. Rognetta, which do not claim the aid of *hyposthenisant* or *antiphlogistic* medication. Its object is to diminish or destroy the morbid excess of organic force, and thus to re-establish the functions. It consists of several elements, whereof some are mechanical, and act indirectly upon the dynamism, that is to say, by removing mechanically a certain quantity of stimulus, of blood, heat, electricity, &c., by depletion, abstinence, bathing, cold applications, &c. The others are dynamics, and act directly by assimilation upon the functional force, or rather upon the ganglionic system which represents it; they lower the degree of this force, and if it be above the normal type, they bring it back to its natural point, and re-establish the functions. If their use is still continued, the vitality is brought still lower, and the symptoms are manifested of true asthenia. These last means offer the advantage of modifying directly the erethism of the organs, without taking away any of the substance; it is thus the counter-stimulants act, of which great use is made in the hypersthenic affections of the eye, (tartar emetic, nitrate of potass, calomel, belladonna, sulphate of magnesia, &c.) If there is a plethora, these means alone will be insufficient, for they combat only the dynamic condition, leaving the congestion, which, although now passive, is sufficient to prevent the resolution of the disease, and even to reproduce the dynamic phenomena which the remedies had succeeded in dissipating. It is always, therefore, with evacuations of blood that the antiphlogistic treatment of acute affections of the eye should commence, if attended with plethora. A hypersthenic disease may exist without this last circumstance, and then bleeding would be useless, if not hurtful; such is the case in certain scrofulous, chlorotic, and syphilitic diseases of the eye.

In these circumstances, the disease resists bleeding, although its nature is one of excitation, and it often yields, as if by enchantment, under the influence of direct hyposthenisants, as iodine, iron, mercury, secale cornutum, nitrate of potass, &c. It is easy to understand, then, how bloodletting, which in general is useful in inflammatory diseases, may prove inefficacious in certain inflammations, without the aid of certain direct hyposthenisants. Purulent ophthalmia, retinitis, iritis, choroiditis, are examples. In such cases, it is a common practice to prescribe hyposthenisants in large doses immediately after bleeding, which does not prevent a recurrence several times to this remedy, if needs be. The remedies in question answer so much the better after bloodletting, that they keep up, prolong, and augment its good effects.

Such are our author's views respecting antiphlogistics, or hyposthenisants.

3. Theoretically, by *revulsion* is understood an artificial action on some point more or less remote from the part diseased, with the view of displacing or causing the disappearance of the disease. This action consists sometimes in an artificial disease, such as a burn with a red-hot iron, boiling water, the potential cautery, a blister, urtication, an eruptive ointment, &c.; sometimes in the use of medicines called evacuants, and of which the object is to promote secretions, or to draw off a quantity, more or less copious, of the animal fluids. The meaning of all this is, that we can, by the aid of those means, displace a disease—make it pass from the inside to the outside, from the upper regions of the body to the lower, from the lungs to the skin of the arm or leg, from the head to the intestines, from the eyes to the nape or the arms.

Dr. Rognetta remarks, that a slight reflection will show that, in principle, the mode of reasoning is no other than that of the humoral pathology. He rejects, then, the revulsive theory, and ridicules with justice the notion of revulsive bloodlettings, while he admits the utility of bloodletting as a mechanical means, diminishing the quantity of blood, and, as a dynamical, its indirect action being to diminish the stimulus of the circulation. Neither has he any greater respect for derivatives, such as cupping, the warm bath, rubefacients and vesicatories, as derivatives. Cupping acts exactly as venesection, the warm bath deprives the body of part of its caloric, while blisters act, he thinks, by being absorbed. Locally, he says, the action of a blister is insignificant. But the cantharides are absorbed, they act on the dynamism of the disordered organ, they hyposthenize it, and hence the antiphlogistic effect.—Dr. Rognetta holds, that an issue does not act by revulsion. But the continued loss of blood by means of the purulent discharge, weakens the system, and produces a hyposthenizing effect.

Purgatives, according to our author, act only by being absorbed, and benefit not so much by their effect as evacuants as by their directly sedative influence. That they will act by being absorbed, as when castor oil is rubbed into the skin, we do not deny, but we conceive it to be beyond a doubt that they purge, not merely from their being taken up from the bowels and conveyed into the tide of the blood, but from their immediate action on the intestinal mucous membrane, and hence on the muscular coat.

4. Dr. Rognetta includes all medical applications made to the eyes themselves under the name of *collyria*, and staggers us at the outset by advancing the dogma, that all of them act only by being absorbed, exactly as medicines do when applied to the internal surface of the stomach. Some of them, however, he admits to act mechanically, or even chemically, such as cold water, compression, caustics. Always, and even in the cases now referred to, a dynamic effect is to be recognized ; for potential caustics do not limit their effects to mere burning, but are partly absorbed, and act dynamically. Even cold water, while it subtracts heat, determines a dynamic effect. From these data, he draws the conclusion, that the efficaciousness of a collyrium is in proportion to its being absorbed, or passing into the circulation of the eye.

The doctor exclaims against combining a number of ingredients together in a collyrium, probably of opposite medicinal characters, or which upon being combined produce something opposite in the effects from what is intended. He instances sulphate of zinc and opium, acetate of lead and camphorated spirit of wine, acetic acid and spirit of wine, which being combinations of stimulants and sedatives, produce compounds whose actions are either null, or opposed to what is intended. We perfectly agree with Dr. Rognetta, that collyria should be as simple as possible. He arranges collyria under six heads : viz. 1, gaseous ; 2, fluid ; 3, soft or unctuous ; 4, dry or pulverulent ; 5, metallic, which contain either silver, mercury, copper, lead, zinc, alum, hydrocyanic acid, nitre, or common salt ; 6, vegetable and animal. What hydrocyanic acid has to do among the metals we cannot divine.

Along with many useful hints in his discussion of these various local applications to the eyes, we encounter not a few prejudices and absurdities ; such as, that a warm collyrium will do harm in the greater number of cases—that the application of fluid collyria by means of a camel-hair pencil is painful, from the mechanical effect of the instrument—and that a better way is to cover the finger with a bit of rag, dip it into the collyrium, carry it gently along the inside of the eyelids, and then leaving the rag between the eyelids and in contact with the eye !

With respect to the metallic collyria, he recommends them to be used in the fluid forms, and very weak, so that they may be the more readily absorbed. For instance, he limits the collyrium of nitrate of silver to three or four grains of the salt in an ounce of water, but recommends chiefly from one to three grains, the solution being used as a fomentation. In this degree of strength, he asserts it is absorbed by the eye, and by this means acts beneficially, not in mere conjunctivitis only, but in diseases of the choroid, and even of the retina. In chronic ophthalmia, his practice is, to agitate a stick of lunar caustic for a few instants in a basin of water, till it becomes a little turbid, and to let the patient use this as a lotion for the eyes and face with a sponge. He states this to be attended with great advantage. In like manner with regard to sulphate of copper in cases of chronic blepharitis, he pours a few drops of a concentrated solution of it into a basin of water, and with this directs the patient to bathe the eyelids and the face, morning and evening, thus employing a sort of mineral water, the quantity of which makes up for the weakness of the saline ingredient. The absorption of it, the doctor thinks, is therefore easily effected, while if strong, it could not be tolerated, and could do no good. The weak solutions act upon all the vessels of the face, contributing to the vascularity of the eyelids and conjunctiva, and prove highly beneficial.

Dr. Rognetta, on the whole, seems well acquainted with chemistry; but sometimes makes a slip, as when he tells us, (p. 41,) that in blue ointment the mercury is oxidized. He might have learned long ago from Orfila, that it is merely mechanically subdivided, with scarcely a trace of oxide.

Corrosive sublimate, he recommends, not only in solution, but in salve, one part of the salt to be dissolved in distilled water and mixed with sixteen parts of fatty matter. This, we conceive, would be too irritating to be applied to the eye.

It is somewhat surprising, that Dr. Rognetta does not seem to be aware of the danger of using saturnine applications to the eyes.

He recommends highly as a collyrium, or drop for the eye, a mixture of equal parts of a saturated solution of alum, and a saturated solution of sulphate of zinc. This, it seems, has succeeded well in the hands of M. Clot-Bey, in the treatment of purulent ophthalmia in Egypt.

A bread and milk poultice, moistened on the surface with a strong solution of nitre, is highly recommended by Dr. Rognetta as a sedative in cases of ophthalmia, attended with pain and heat.

We regret he seems so little aware of the admirable effects of belladonna, employed in the form of a collyrium, doing little more than naming it along with the other vegetable substances. In our experience, few cases of scrofulous ophthalmia have resisted the careful use of a belladonna collyrium, and in many other varieties of inflammation of the eyes it proves scarcely less useful.

We have thus given a pretty full account of Dr. Rognetta's therapeutical principles, because, as we have already hinted, it is in these, much more than in his description of diseases, that his work differs from other ophthalmological treatises. Were we called upon to sum up in a few general conclusions the substance of his therapeutics, we fear one of these conclusions would be this, that in eye-practice, not less than in the treatment of other organs, many—many remedies, of the action of which we know nothing, are every day employed against diseases, the nature of which is equally unknown. This is a truth which, however humiliating, should never damp in the smallest degree the zeal of the honest and observant practitioner of the healing art. The *modus operandi* of medicines is almost wholly unknown—the fact of their being beneficial in many cases cannot be a matter of doubt to any man the eyes of whose understanding are opened, and blinded neither by prejudice nor self-conceit. To throw medicines aside because we cannot explain the mode in which they act, would be the height of folly, and almost as foolish is the attempt to arrange them within a very limited number of artificial divisions, such as those adopted by the author before us. We should much rather have no classification of remedies at all than that which arranges them into the two divisions of excitants and sedatives.—We should much rather call quina a specific, mercury a specific, sulphur a specific, iodine a specific, for the several diseases which these remedies are known to cure, because we should thereby declare a fact, which we should not do by calling any of them an excitant or a sedative.

What is the grand practical result of the revolution in the classification of remedies, attempted by Giacomini? Almost nothing. We find Dr. Rognetta raising his voice against putting a little alcohol into a lead collyrium, because, forsooth, the alcohol is a stimulant, and would undo the sedative effect of the lead—or of combining opium with calomel, in iritis, because the hypersthenisant properties of the opium would counteract the hypersthenisant ones of the calomel. But this is all. The same remedies essentially are employed by our author as are used by other practitioners, or if his new therapeutics lead him to any practice very different from the common, it is to some such absurdity as attempting to cure iritis and other internal ophthalmiæ by atropism, (p. 242,) that is to say, by poisoning the patient with belladonna—a prank supremely useless, even when it does not prove dangerous.

The division of all diseases into sthenic and asthenic, and of all remedies into hypersthenisant and hyposthenisant, is absurd, and fit to take its place only in the childish brain of some quixotical water-curer, who, holding "the leading paramount indication in the treatment of any given disease, to be either to depress excited action, or to exalt depressed action," tells us, that "one single remedy, capable of being graduated in its doses, so as to exercise every degree of sedation or stimulation respectively, is calculated, under favorable circumstances, to operate against the whole host of maladies, and to supersede, or be backed against, the whole list of medicines,"* which remedy is water.

Barring his theoretical notions about the action of remedies, we can scarcely give Dr. Rognetta too much credit for his book, which, as we stated at the outset, is both well written, and rich in characteristic descriptions of disease, full of valuable facts, and rendered interesting by the reasonings of an acute and lively mind. With one short extract, explanatory of Dr. Rognetta's mode of regarding some of the most important disorders of the eye, we shall take our leave of him.

"The apparatus of vision may be considered as a direct emanation, a prolongation of the brain. In an anatomical point of view, the eye is, in fact, a sort of little brain. Like the encephalon, it presents a protecting osseous case (orbit), completed by a fibro-membranous apparatus (eyelids). Like the brain, it has a fibrous envelop (sclerotica), another which is vascular and serous (choroid), and lastly, an essential organic part, which is medullary or nervous (retina). Let us observe this portion of the cerebral substance, which is continued within the sheath of the optic nerve, and expands over the bottom of the eye; this considerable artery, which penetrates it (arteria centralis), and which imitates the basilar artery of the brain; this prodigious quantity of nerves which surround the eye, and the branches of which enter into its interior; and, lastly, this magnificent tree of arterial vessels, which bathe with blood the eye, the orbit, and all the neighbouring parts, as the carotid does the parts to which it is distributed; all this leads naturally to the conclusion, that one of the principal sources of the diseases of the eye is in the brain itself, or at least in the constituent elements of the eye, coming from the interior of the cranium. Thus it is that I see, in a great number of these diseases, affections depending on a certain morbid state of the encephalon, and I can every day congratulate myself on the practical results accruing from this manner of contemplating my subject." (p. 1.)

* Balbirnie's Philosophy of the Water-Cure, p. 100.

